# Late-Onset Calorie Restriction Improves Lipid Metabolism and Aggravates Inflammation in the Liver of Old Wistar Rats

**DOI:** 10.3389/fnut.2022.899255

**Published:** 2022-05-23

**Authors:** Ana Teofilović, Miloš Vratarić, Nataša Veličković, Danijela Vojnović Milutinović, Aleksandra Mladenovic, Milica Prvulovic, Ana Djordjevic

**Affiliations:** ^1^Department of Biochemistry, Institute for Biological Research “Siniša Stanković” – National Institute of Republic of Serbia, University of Belgrade, Belgrade, Serbia; ^2^Department of Neurobiology, Institute for Biological Research “Siniša Stanković” – National Institute of Republic of Serbia, University of Belgrade, Belgrade, Serbia

**Keywords:** calorie restriction, lipid metabolism, inflammation, liver, aging

## Abstract

Aging is a progressive process that could disturb metabolic homeostasis in the liver *via* ectopic lipid accumulation, oxidative stress, and deterioration of inflammatory response. Although calorie restriction (CR) is recognized as beneficial for life span and health span prolongation, it is still unclear how late-onset CR, characterized by late beginning and short duration, affects age-related processes. The aim of this study was to examine how late-onset CR-induced metabolic adjustments impact lipid status and inflammation in the liver of old rats. The experiments were conducted on aging male Wistar rats fed *ad libitum* (AL) or exposed to late-onset CR (60% of AL daily intake) from 21st to 24th month. The results showed that late-onset CR reduces body weight, visceral adipose tissue and liver mass, and triglyceride levels when compared to old animals on AL diet. The ameliorating effects of CR on lipid metabolism include increased activity of AMP-activated protein kinase, suppressed *de novo* fatty acid synthesis, stimulated β-oxidation, decreased lipotoxicity, and limited triglyceride synthesis and packaging in the liver. Restricted diet regime, however, does not improve expression of antioxidant enzymes, although it leads to progression of age-related inflammation in the liver, partially through lower corticosterone concentration and decreased activation of glucocorticoid receptor. In conclusion, late-onset CR is able to restore age-related imbalance of lipid metabolism in the liver, but has a negative impact on hepatic inflammatory status, implying that the type of diet for older individuals must be balanced and chosen carefully with appropriate duration and start point.

## Introduction

Aging is a natural and progressive process and a major risk factor for development of neurodegenerative and cardiovascular diseases, type 2 diabetes, metabolic syndrome, and tumors (1, 2). One of the most commonly investigated experimental approaches for life span and health span increase and attenuation of age-associated disorders is calorie restriction (CR). At the molecular level, CR activates lipolysis in adipose tissue, stimulates mitochondrial oxidation of fatty acids (FA), reduces dyslipidemia, increases insulin sensitivity, and regulates immune response (3). A key role in mediating the effects of CR on age-related diseases is played by the liver, as it is crucial for the maintenance of energy homeostasis (4).

The aging process is commonly accompanied by visceral adiposity and disturbed lipid homeostasis in the liver. Elevated influx of FA from adipose tissue, stimulation of *de novo* lipogenesis, increased production of very-low-density lipoproteins (VLDL), and lower rate of β-oxidation lead to the progression of hepatic lipotoxicity, steatosis, and dyslipidemia. A well-characterized sensor of disrupted energy balance, whose regulatory potential declines with aging, is the AMP-activated protein kinase (AMPK) (5, 6). Increased AMPK activity induced by CR could be a relevant mediator in lipid metabolism restoration through stimulation of ATP-producing processes (7). AMPK inhibits the activity of one of the key enzymes of *de novo* lipogenesis, acetyl-CoA carboxylase (ACC) by its phosphorylation at Serine 79. Reduced availability of ACC product, malonyl-CoA, results in activation of carnitine palmitoyl-transferase 1 (CPT1), which leads to increased transport of FA into the mitochondria and elevated intensity of β-oxidation (8). AMPK can also phosphorylate and stimulate peroxisome proliferator-activated receptor gamma coactivator 1α (PGC-1α), a transcriptional activator of CPT1, which additionally contributes to FA elimination (9).

Disturbed metabolic balance and lipid accumulation during aging are both potent triggers for the development of chronic inflammation in the liver. Excess of FA could directly stimulate proinflammatory Toll-like receptor 4 (TLR4), while both FA and lipid species can activate proinflammatory transcriptional regulator nuclear factor kappa B (NFκB), which upregulates the expression of tumor necrosis factor α (TNFα), interleukins, and chemokines (10, 11). Age-associated oxidative stress, a result of increased production of reactive oxygen species (ROS) and decreased expression and activity of antioxidant enzymes, is also a well-described activator of NFκB and downstream inflammatory signaling. Although the early initiated CR is recognized as a potent factor in improving oxidative stress, its effect on redox balance during aging is complex and depends on species, sex, tissue, and duration of limited calorie intake (12, 13).

The age-induced inflammation is usually counterbalanced by increased systemic concentrations of glucocorticoid hormones (14). It was shown that energy depletion related to CR additionally elevates the level of glucocorticoids that could override inflammatory processes and suppress immune response (15). The anti-inflammatory properties of glucocorticoids are achieved by activating the glucocorticoid receptor (GR), a transcriptional factor that negatively regulates the expression of NFκB and other proinflammatory genes. Tissue-specific action of glucocorticoids is more complex, as their intracellular concentration depends on hormone regeneration by the enzyme 11β-hydroxysteroid dehydrogenase type 1 (11β-HSD1) and glucocorticoid clearance regulated by 5α and 5β-reductases (16, 17).

Although a large number of studies point out the beneficial effects of CR, its onset, duration, and percentage of restriction are recognized as important determinants of restrictive diet welfare. Late-onset and short duration of CR provoke significantly lower beneficial cognitive response and even worsen age-related frailty as compared to lifelong restriction started at young age (18, 19). As it is not clear how CR started at old age affects disturbed metabolic balance caused by aging, the aim of this study was to examine the impact of metabolic alterations induced by late-onset CR on lipid status and inflammation in the liver of old male Wistar rats. To that end, we analyzed the level of stimulatory phosphorylation of the energy sensor AMPK, expression of enzymes and transporters involved in *de novo* lipogenesis, FA oxidation, and triglyceride packaging. The inflammatory status was examined through gene expression of *Tlr4* and *Tnf*α, and through protein level and subcellular distribution of NFκB. Protein levels of antioxidant enzymes, glucocorticoid prereceptor metabolism, and GR expression in the liver of old rats upon CR were also assessed.

## Materials and Methods

### Animals and Treatment

In the beginning of the experiment, male Wistar rats were divided into three groups (*n* = 8 per group). Two groups had *ad libitum* (AL) access to the standard commercial food until they were 6 (6 mAL) or 24 months old (24 mAL). The third group (24 mCR) had unlimited access to food till the 21st month of age, which was followed by a restricted diet regime from 21 to 24 month (late-onset CR) when the animals received 60% of average AL daily food intake. Animals have been acclimated to CR, as restricted diet regime was gradually introduced by reducing the daily average food intake by 10% every 2 days; so after 7 days, food consumption decreased by 40%. The detailed composition of used commercial food (Veterinary Institute Subotica, Subotica, Serbia) was previously published (20). The animals were housed under standard conditions (22°C, 12 h light and dark cycles, constant humidity, free access to water, housed two-three per cage). All the procedures were in compliance with the European Communities Council Directive (2010/63/EU) and were approved by the Ethical Committee for the Use of Laboratory Animals of the Institute for Biological Research Siniša Stanković – The National Institute of Republic of Serbia, University of Belgrade (reference number 02-12/17).

### Tissue Collection, Blood Plasma Preparation, and Determination of Blood Parameters

After overnight fasting, animals were anesthetized with an intraperitoneal injection of Zoletil 100 (75 mg/kg) and sacrificed. Blood was rapidly collected from the trunk into polypropylene tubes. Serum was isolated after 30 min of coagulation at room temperature and centrifuged at 2,000 × *g* for 15 min. Collected sera were stored at –70°C for later processing. Glucose concentrations in serum were measured by using a commercially available reagent (OSR6121, Beckman Coulter, Brea, CA, United States) on Olympus AU400 (BLOCK Scientific, North Bellport, NY, United States). The concentrations of serum free fatty acids (FFAs) were assessed by a commercially available kit (FA115, Randox Laboratories Ltd., Crumlin, United Kingdom) and serum triglycerides (TG) were analyzed with triglycerides reagent (Code 12528, Biosystem, Barcelona, Spain). Both measurements were performed on the semi-automatic biochemistry analyzer Rayto 1904-C (Rayto, Shenzhen, China).

After blood collection and transcardial perfusion, livers and retroperitoneal and perirenal depots of visceral adipose tissue (VAT) were carefully excised, weighed, and stored in liquid nitrogen until further analysis.

### Preparation of Whole-Cell Extract

Frozen livers from individual animals were weighed and homogenized with a glass–Teflon homogenizer in five volumes (w/v) of ice-cold RIPA buffer [50 mM Tris-HCl, pH 7.2, 1 mM EDTA-Na_2_, 150 mM NaCl, 0.1% SDS, 1% Nonidet P-40, 0.5% sodium deoxycholate, 2 mM dithiothreitol (DTT), protease and phosphatase inhibitors]. Homogenates were sonicated (5 s/30 s pause/5 s/30 s pause/5 s, 1A, 50/60 Hz, Hielscher Ultrasound Processor, Teltow, Germany), incubated for 60 min on ice with continuous agitation and vortexing, and centrifuged (16,000 × *g*, 20 min, 4°C, Eppendorf 5804/R, Hamburg, Germany). The supernatants were used as the whole-cell extracts.

### Preparation of Cytoplasmic, Nuclear, Microsomal, and Mitochondrial Fractions of the Liver

After thawing, livers from individual animals were weighed and homogenized with Janke-Kunkel Ultra Turax (30 s/30 s pause/30 s) in four volumes (w/v) of ice-cold homogenization buffer (20 mM Tris-HCl pH 7.2, 10% glycerol, 50 mM NaCl, 1 mM EDTA-disodium, 1 mM EGTA Na_2_, 2 mM DTT, protease and phosphatase inhibitors). The homogenates were filtered through gauze and centrifuged (2,000 × *g*, 15 min, 4°C). The resulting supernatants (S1) were further processed to generate cytoplasmic, microsomal, and mitochondrial fractions, while the pellets (P1) were used to obtain nuclear fractions. The S1 were centrifuged (14,000 × *g*, 30 min, 4°C, Eppendorf 5804/R, Hamburg, Germany). Resulting supernatants (S2) were further processed to generate cytoplasmic and microsomal fractions, while the pellets (P2) were resuspended in one volume of homogenization buffer, which contain 0.05% Triton X-100 and were used as a mitochondrial fraction. The S2 were ultracentrifuged (200,000 × *g*, 90 min, 4°C, Beckman L7-55, Brea, CA, United States) and the final supernatants were used as cytoplasmic fractions. The final pellets were resuspended in 100 mM Na-pyrophosphate buffer (pH 7.4) and centrifuged (200,000 × *g*, 60 min, 4°C, Beckman L7-55, Brea, CA, United States) for the preparation of microsomal fractions. The microsomal pellets were suspended in a storage buffer (50 mM KPO_4_ pH 7.4, 0.1 mM EDTA-disodium, 10% glycerol, and 0.1 mM DTT Hielscher Ultrasound Processor, Teltow, Germany), sonicated (5 s/30 s pause/5 s/30 s pause/5 s, 1A, 50/60 Hz, Hielscher Ultrasound Processor, Teltow, Germany), and used as microsomal fractions. For nuclear fractions preparation, P1 from the first centrifugation were washed twice in HEPES buffer (25 mM HEPES pH 7.6, 1 mM EDTA-disodium, 1 mM EGTA-disodium, 10% glycerol, 50 mM NaCl, 2 mM DTT, protease and phosphatase inhibitors like in homogenization buffer, Eppendorf 5804/R, Hamburg, Germany, Eppendorf 5804/R, Hamburg, Germany) by centrifugation (4,000 × *g*, 10 min, 4°C). The resulting pellets were suspended in one volume of NUN buffer (25 mM HEPES, pH 7.6, 1 M Urea, 300 mM NaCl, 1% Nonidet P-40, protease and phosphatase inhibitors). After incubation on ice for 90 min with continuous agitation and frequent vortexing, suspensions were centrifuged (8,000 × *g*, 10 min, 4°C, Eppendorf 5804/R, Hamburg, Germany). The resulting supernatants were used as nuclear fractions. All steps were conducted at 0–4°C and all samples were aliquoted and stored at −70°C.

### Preparation of Membrane Fraction of the Liver

Parts of frozen livers from individual animals were weighed and homogenized with a glass-Teflon homogenizer in ten volumes (w/v) of ice-cold DEA buffer (0.25% diethyl amine, 100 mM NaCl, protease and phosphatase inhibitors) and centrifuged (100,000 × *g*, 30 min, 4°C, Beckman L7-55, Brea, CA, United States). The pellets were homogenized in the same volume of 1% Triton buffer (1% Triton, 150 mM NaCl, 50 mM Tris-HCl pH 7.4, 2 mM EDTA, protease and phosphatase inhibitors) with a glass-Teflon homogenizer. Homogenates were put through a 23-gauge needle, incubated for 30 min on ice, and centrifuged (100,000 × *g*, 30 min, 4°C, Beckman L7-55, Brea, CA, United States). The supernatants were used as membrane fractions. Protein content of all fractions was determined by the method of Spector (21) using bovine serum albumin as a standard.

### Corticosterone Assay

Liver CORT concentrations were determined in the cytoplasmic fractions by a Corticosterone High Sensitivity EIA kit (Corticosterone HSEIA, AC-15F1, Immunodiagnostic Systems Ltd., Boldon Colliery, United Kingdom), according to the manufacturer’s instructions. The standards and samples were measured in duplicate. The plates were read at 450 and 650 nm on the Multiskan Spectrum (Thermo Electron Corporation, Waltham, MA, United States). The hormone concentrations were determined using the 4PL curve-fitting method (Graph-Pad Prism 5, GraphPad Software, Inc., San Diego, CA, United States) and are given as ng/mg of proteins.

### Western Blot Analysis

The samples were boiled in Laemmli’s buffer and 40 μg proteins of cytoplasmic, microsomal, and whole cell extracts and 60 μg proteins of nuclear, mitochondrial, and membrane fractions were resolved on 8 or 10% sodium dodecyl sulfate-polyacrylamide gels. After protein transfer from the gels to polyvinylidenedifluoride membranes (Immobilon-FL, Merck Millipore, Burlington, MA, United States), membranes were incubated overnight at 4°C with the following primary polyclonal antibodies: anti-AMPKα1/2 (sc-25792, 1:500), anti-CD36 (sc-7309, 1:1,000), anti-CPT1 (sc-139482, 1:500), anti-IκB-α (sc-371, 1:250), anti-phospho-IκB-α (Ser32) (sc-2859s, 1:250), anti-NFκB/p65 (sc-372, 1:1,000), anti-Hexose-6-phosphate dehydrogenase (H6PDH) (sc-67394, 1:500), and anti-GR (sc- 8992, 1:250), all purchased from Santa Cruz Biotechnology (Dallas, TX, United States). Anti-phospho-AMPKα1/2 (Thr 172) (2535, 1:1,000), anti-ACC (3676, 1:1,000), and anti-phospho-ACC (Ser70), (1:1,000) were purchased from Cell Signaling Technology (Danvers, TX, United States), while anti-PGC1α (ab54481, 1:250), anti-Catalase (ab16731, 1:2,000), anti-SOD1 (ab13498, 1:2,000), anti-SOD2 (ab13533, 1:2,000), and anti-11β-HSD1 (ab393364, 1:1,000) were obtained from Abcam (Cambridge, United Kingdom).

As the proteins of interest were detected in the different fractions of the hepatocytes, constitutively expressed proteins specific for each fraction were used as equal load controls. Namely, anti-β-actin (ab8227, Abcam, 1:10,000) was used as the loading control for cytoplasmatic fraction and whole cell extract, while anti-Hsp60 (sc-13115, Santa Cruz, 1:1,000), anti-Lamin B1 (sc-374015, Santa Cruz, 1:1,000), and anti-Calnexin (ab22595, Abcam, 1:2,000) were used for mitochondrial, nuclear, and microsomal fraction, respectively. Ponceau S was used as the loading control for plasma membrane fraction. Membranes were subsequently washed and incubated for 90 min with horseradish peroxidase conjugated secondary antibodies, goat anti-rabbit IgG (ab6721, Abcam, 1:20,000), or rabbit anti-mouse IgG (ab97046, Abcam, 1:30,000). The immunoreactive protein bands were visualized by chemiluminiscent (ECL) method using iBright CL1500 (Thermo Fisher Scientific, Waltham, MA, United States) and quantitative analysis was performed by iBright software.

### RNA Isolation and Reverse Transcription

Total RNA was isolated from the liver using TRIreagent^®^ (AmBion, Life Technologies, Austin, TX, United States). Concentrations of isolated RNA were quantified on NanoPhotometer N60 (Implen, Munich, Germany) by reading the optical density at 260 nm, while the quality and integrity of RNA was confirmed on 2% agarose gel. For the synthesis of complementary DNA (cDNA), Applied Biosystems High-Capacity cDNA reverse Transcription Kit (Thermo Fisher Scientific, Waltham, MA, United States) was used according to the manufacturer’s instructions. The cDNA was stored at −70°C until analysis.

### Real-Time Polymerase Chain Reaction

The expression of glucokinase (*Gck*), *Tlr4*, stearoyl-CoA desaturase 1 (*Scd1*), microsomal triglyceride transfer protein (*Mttp*), and apolipoprotein B-100 (*Apo B-100*) genes was analyzed using Power SYBR^®^ Green PCR Master Mix (Applied Biosystems, Thermo Fisher Scientific, Waltham, MA, United States). Specific primers (Metabion, Planegg, Germany) for *Gck* (forward 5′-AGA TGC TAT CAA GAG GAG AG-3′, reverse 5′-ACA ATC ATG CCG ACC TCA CAT-3′), *Tlr4* (forward 5′-ATC ATC CAG GAA GGC TTC CA-3′, reverse 5′-GCT AAG AGG GCG ATA CAA TTC-3′), *Scd1* (forward 5′-TGG TGC TCT TTC CCT GTT TGC-3′, reverse 5′-TGG GCT TTG GAA GGT GGA CA-3′), *Mttp* (forward 5′-AAG GCC AAT ATG GAC ATC CAG GTT-3′, reverse 5′-TGG TTA TTA CCA CAG CCA CCC GAT-3′), and *Apo B-100* (forward 5′-AGT CTA CTG GAA GCC ATG AAG GG-3′, reverse 5′-AAT CTG CTG AGG AAG CCT GCT CA-3′) were used for selective amplification. Quantitative normalization of cDNA in each sample was performed using β*-actin* (forward 5′-GAC CCA GAT CAT GTT TGA GAC C-3′, reverse 5′-AGG CAT ACA GGG ACA ACA CA-3′) as the endogenous control. Thermal cycling conditions were 2 min incubation at 50°C, 10 min at 95°C followed by 40 cycles of 95°C for 15 s, and 60°C for 60 s. Quantification of *Tnf*α (Rn01525859_g1) and *5*α*-reductase* (Rn00567064_m1) genes expression was performed using TaqMan^®^ gene expression FAM-labeled probe set (Applied Biosystems, Thermo Fisher Scientific, Waltham, MA, United States). Quantitative normalization of cDNA in each sample was performed using β*-actin* (Rn00667869_m1) as the endogenous control. Thermal cycling conditions were 2 min incubation at 50°C, 10 min at 95°C followed by 60 cycles of 95°C for 15 s, and 60°C for 60 s. All real-time PCR reactions were performed in duplicate using Quant Studio™3 (Applied Biosystems, Thermo Fisher Scientific, Waltham, MA, United States). Relative quantification of gene expression was performed using the comparative 2^–ΔΔCt^ method. The same cDNA simple was used as the calibrator on each plate. The results were analyzed by Quant Studio™ Design & Analysis v1.3.1 (Applied Biosystems, Thermo Fisher Scientific, Waltham, MA, United States) with a confidence level of 95% (*p* ≤ 0.05).

### Statistical Analysis

All data are given as means ± SEM (*n* = 8). The data were tested for normality by Shapiro–Wilk test. Normally distributed data were analyzed by one-way ANOVA followed by Bonferroni *post-hoc* test. Data with deviation from normal distribution were analyzed by Kruskal–Wallis H test followed by Mann–Whitney U *post-hoc* test with Bonferroni correction. A *p*-value < 0.05 was considered statistically significant. Statistical analyses were performed by the STATISTICA 12 software (StatSoft, Inc., Tulsa, OK, United States).

## Results

### The Effects of Late-Onset Calorie Restriction on Morphological and Biochemical Parameters

Body weight, liver, and VAT mass were significantly increased in aged rats on AL diet as compared to adult rats (24 mAL vs. 6 mAL, *p < 0.05 for body and liver mass; ^***^p < 0.001 for VAT mass), whereas CR treatment reduced these parameters to control levels (24 mCR vs. 24 mAL, ^#^*p* < 0.05 for body and liver mass; ^###^*p* < 0.001 for VAT mass). The VAT-to-body ratio was changed in the same manner (increased, 24 mAL vs. 6 mAL, ^***^p < 0.001; decreased, 24 mCR vs. 24 mAL, ^###^*p* < 0.001), but liver-to-body ratio did not show any changes (Table 1).

**TABLE 1:** The effects of late-onset calorie restriction on morphological and biochemical parameters.

	6 mAL	24 mAL	24 mCR
Body mass (g)	403.75 ± 5.96	510.00 ± 28.45 *	423.75 ± 19.36 ^#^
Mass of liver (g)	9.88 ± 0.28	11.66 ± 0.61 *	9.74 ± 0.45 ^#^
Liver to body mass ratio (x1,000)	24.48 ± 0.69	23.88 ± 1.55	23.04 ± 0.64
Mass of VAT (g)	5.40 ± 0.36	13.25 ± 1.70 ***	5.97 ± 0.82 ^###^
VAT to body mass ratio (x1,000)	13.37 ± 0.84	26.08 ± 2.24 ***	13.77 ± 1.35 ^###^
Glucose (mmol/l)	8.51 ± 0.28	6.51 ± 0.58 *	6.70 ± 0.45 ^$^
*Gck* mRNA (fold change)	1.00 ± 0.19	6.00 ± 1.51**	9.53 ± 1.77^$$$^
FFA (mmol/l)	1.25 ± 0.20	5.33 ± 1.61 *	2.91 ± 1.10
TG (mmol/l)	0.73 ± 0.09	1.35 ± 0.14 *	0.85 ± 0.11 ^#^
Liver CORT (ng/mg)	0.33 ± 0.02	0.57 ± 0.09 *	0.35 ± 0.03 ^#^

Body weight, liver and visceral adipose tissue (VAT) mass, concentration of blood glucose, free fatty acids (FFA) and triglycerides (TG), liver corticosterone (CORT), and glucokinase (Gck) mRNA level were measured in 24th-month-old male Wistar rats who were on ad libitum (AL) diet or on calorie restriction (CR) from 21st to 24th months. All data are presented as means ± SEM (n = 8). Different symbols denote significant differences between aged rats on AL diet and adults (24 mAL vs. 6 mAL, *p < 0.05, **p < 0.01, ***p < 0.001), old rats on CR and on AL diet (24 mCR vs. 24 mAL, ^#^p < 0.05, ^###^p < 0.001), and old rats on CR and adults fed AL (24 mAL vs. 6 mAL, ^[dollar]^p < 0.05, ^$$$^p < 0.001).

Serum glucose concentration was decreased in aged rats regardless of the applied diet in comparison with adults (24 mAL vs. 6 mAL, *p < 0.05; 24 mCR vs. 6 mAL, ^$^*p* < 0.05; Table 1). This was in agreement with elevated *Gck* mRNA levels in both groups of older rats compared to adults (24 mAL vs. 6 mAL, ^**^p < 0.01; 24 mCR vs. 6 mAL, ^$$$^*p* < 0.001; Table 1). FFAs in the serum were elevated in aged rats on AL diet as compared with adults (24 mAL vs. 6 mAL, *p < 0.05; Table 1), while CR treatment did not provoke any differences. Serum triglyceride level was also significantly increased in aged rats (24 mAL vs. 6 mAL, *p < 0.05), whereas CR treatment reduced this parameter (24 mCR vs. 24 mAL, #*p* < 0.05).

### Late-Onset Calorie Restriction Improves Lipid Status in the Liver of Old Rats

**FIGURE 1:**
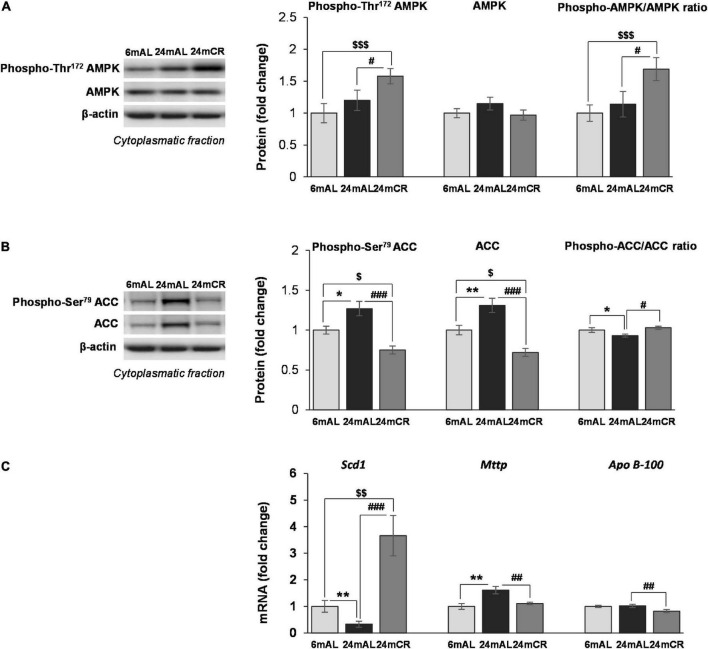
The levels of phosphorylated and total AMPK and ACC proteins and other mediators of lipid synthesis in the liver. Representative Western blots and relative quantification of phospho (Thr172) and total AMPK **(A)** and phospho (Ser79) and total ACC **(B)** in the cytoplasmatic fraction of the liver of adults (6 mAL), aged rats on AL diet (24 mAL), and old rats on CR (24 mCR). β-Actin was used as the loading control for cytoplasmatic fraction. Quantification of *Scd1*, *Mttp*, and *Apo B-100* mRNA levels **(C)** in the liver was done relative to the amount of β-actin. All values are given as means ± SEM (*n* = 8). Asterisk indicates a significant difference between aged rats on AL diet and adults (24 mAL vs. 6 mAL, *p < 0.05, **p < 0.01), hashtag indicates a difference between old rats on different diet regimes (24 mCR vs. 24 mAL, ^#^*p* < 0.05, ^##^*p* < 0.01, ^###^*p* < 0.001), and symbol $ denotes a difference between old rats on CR and adults fed AL (24 mCR vs. 6 mAL, ^$^*p* < 0.05, ^[*dollar*][*dollar*]^*p* < 0.01, ^$$$^*p* < 0.001).

In the liver of aged rats on AL diet regime, neither energy sensor AMPK protein expression, nor its stimulatory phosphorylation on Threonine 172 were changed compared with adults. The level of phosphorylated form of AMPK, as well as phospho-AMPK/total AMPK ratio, were elevated in rats after CR treatment compared with animals fed AL (24 mCR vs. 24 mAL, ^ #^*p* < 0.05; 24 mCR vs. 6 mAL, ^$$$^*p* < 0.001; Figure 1A), implying that reduced caloric intake led to increased AMPK activity.

The aging process resulted in enhanced expression of total ACC protein level (24 mAL vs. 6 mAL, ^**^p < 0.01) and its phosphorylated form on Serine 79 (24 mAL vs. 6 mAL, *p < 0.05; Figure 1B). Late-onset CR reduced the level of total and phosphorylated ACC as compared with AL diet regime regardless of age (24 mCR vs. 24 mAL,^ ###^*p* < 0.001; 24 mCR vs. 6 mAL, ^$^*p* < 0.05). The ratio of inhibitory phosphorylation to total ACC was decreased in the liver of aged rats in comparison to adult animals (24 mAL *vs.* 6 mAL, *p < 0.05), while attenuation of *de novo* lipogenesis after CR was supported by increment of this ratio in 24 mCR group in regard to 24 mAL group (24 mCR vs. 24 mAL, ^#^*p* < 0.05; Figure 1B). Examination of further steps of FA metabolism showed the reduction of *Scd1* mRNA level in aged rats on AL diet as compared to adults (24 mAL vs. 6 mAL, ^**^p < 0.01; Figure 1C), while CR treatment significantly raised the level of *Scd1* (24 mCR vs. 24 mAL, ^ ###^*p* < 0.001; 24 mCR vs. 6 mAL, ^$$^*p* < 0.01). On the contrary, the gene expression of *Mttp* was increased in aged rats as compared with adults (24 mAL *vs.* 6 mAL, ^**^p < 0.01) and decreased in CR group in regard to aged rats on AL (24 mCR *vs.* 24 mAL, ^##^*p* < 0.01; Figure 1C). This result was in agreement with reduction of *Apo B-100* mRNA level in rats after CR treatment in comparison with old rats on AL diet (24 mCR *vs.* 24 mAL, ^##^*p* < 0.01; Figure 1C) and implied that late-onset CR prevented triglycerides packaging in the liver. As expected, triglycerides in serum were elevated in aged rats as compared with adults (24 mAL vs. 6 mAL, *p < 0.05) and decreased in CR group in regard to aged rats on AL diet (24 mCR vs. 24 mAL, ^#^*p* < 0.05; Table 1).

**FIGURE 2:**
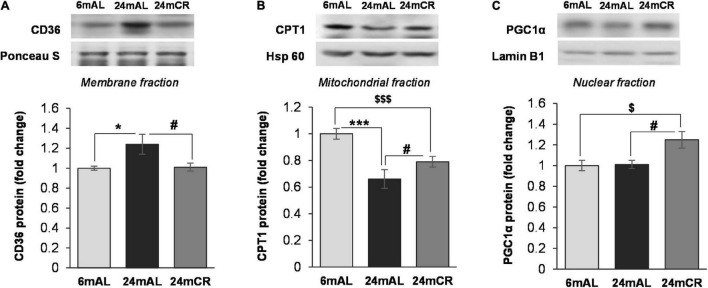
The levels of proteins involved in FA transport and β-oxidation. Representative Western blots and relative quantification of CD36 level in membrane fraction **(A)**, CPT1 level in mitochondrial **(B)**, and PGC1α level in nuclear fraction **(C)** of the liver of adults (6 mAL), aged rats on AL diet (24 mAL) and old rats on CR (24 mCR). Ponceau S, Hsp 60, and Lamin B1 were used as loading controls for membrane, mitochondrial, and nuclear fraction, respectively. All values are given as means ± SEM (*n* = 8). Asterisk indicates a significant difference between aged rats on AL diet and adults (24 mAL vs. 6 mAL, *p < 0.05, ***p < 0.001), hashtag indicates a significant difference between old rats on different diet regimes (24 mCR vs. 24 mAL, ^#^*p* < 0.05), and symbol $ denotes a difference between old rats on CR and adults fed AL (24 mCR *vs.* 6 mAL, ^$^*p* < 0.05, ^$$$^*p* < 0.001).

In accordance with the concentration of FFAs in the serum, the protein level of CD36 transporter in the plasma membrane was increased in old rats on AL diet as compared with adults (24 mAL vs. 6 mAL, *p < 0.05; Figure 2A), while restricted diet regime lowered and equalized its level with adults (24 mCR vs. 24 mAL, ^#^*p* < 0.05; Figure 2A). The intensity of FA β-oxidation was reduced during aging because the CPT1 protein level in the hepatic mitochondrial fraction was reduced in both groups of aged rats as compared to adult animals (24 mAL vs. 6 mAL, ^***^p < 0.001; 24 mCR vs. 6 mAL ^$$$^*p* < 0.001; Figure 2B). However, the applied CR regime partially raised the CPT1 level in comparison with aged animals on a standard diet (24 mCR vs. 24 mAL, ^#^*p* < 0.05; Figure 2B). This finding was supported by increased level of PGC-1α in CR group in regard to groups with AL diet regime (24 mCR vs. 24 mAL, ^#^*p* < 0.05; 24 mCR vs. 6 mAL, ^$^*p* < 0.05; Figure 2C).

### Aging Process Decreases Antioxidant Enzyme Expression, While Late-Onset Calorie Restriction Exacerbates Inflammatory Status in the Liver

**FIGURE 3:**
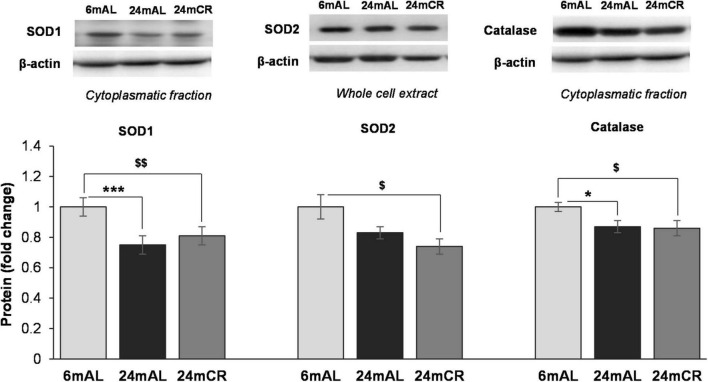
Protein levels of antioxidant enzymes in the liver. Representative Western blots and relative quantification of SOD1 and Catalase in cytoplasmatic and SOD2 protein level in whole cell extract of the liver of adults (6 mAL), aged rats on AL diet (24 mAL), and old rats on CR (24 mCR). β-actin was used as the loading control for cytoplasmatic fraction and whole cell extract. All values are given as means ± SEM (*n* = 8). Asterisk indicates a significant difference between aged rats on AL diet and adults (24 mAL vs. 6 mAL, *p < 0.05, ***p < 0.001) and symbol $ indicates a significant difference between old rats on CR and adults on AL diet (24 mCR vs. 6 mAL, ^$^*p* < 0.05, ^$$^*p* < 0.01).

The aging process, as expected, resulted in a decrease in protein levels of all examined antioxidant enzymes, SOD1, SOD2, and catalase, regardless of the diet regime (24 mAL vs. 6 mAL, ^***^p < 0.001; 24 mCR vs. 6 mAL, ^$$^*p* < 0.01 for SOD1; 24 mCR vs. 6 mAL, ^$^*p* < 0.05 for SOD2; 24 mAL vs. 6 mAL, *p < 0.05; 24 mCR vs. 6 mAL, ^$^*p* < 0.05 for catalase; Figure 3), which implies that late-onset CR did not ameliorate the effects of aging on antioxidant enzymes.

**FIGURE 4:**
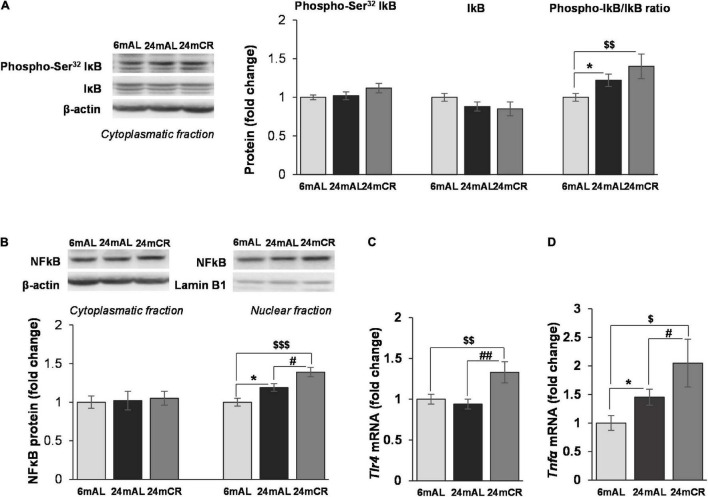
The levels of phosphorylated and total IκB and protein and gene expression of proinflammatory mediators in the liver. Representative Western blots and relative quantification of phospho (Ser32) and total IκB in cytoplasmatic **(A)** and NFκB protein level in citoplasmatic and nuclear fractions **(B)** of the liver of adults (6 mAL), aged rats on AL diet (24 mAL), and old rats on CR (24 mCR). β-actin and Lamin B1 were used as loading controls for cytoplasmatic and nuclear fraction, respectively. Quantification of *Tlr4* **(C)** and *Tnf*α **(D)** mRNA levels in the liver was done relative to the amount of β-actin. All values are given as means ± SEM (*n* = 8). Asterisk indicates a significant difference between aged rats on AL diet and adults (24 mAL vs. 6 mAL, *p < 0.05), hashtag indicates a significant difference between old rats on different diet regimes (24 mCR vs. 24 mAL, ^#^*p* < 0.05, ^##^*p* < 0.01), and symbol $ denotes a difference between old rats on CR and adults on AL diet (24 mCR vs. 6 mAL, ^$^*p* < 0.05, ^$$^*p* < 0.01, ^$$$^*p* < 0.001).

Regarding the inflammation, increased ratio between phosphorylated (at Ser^32^) and total form of NFκB inhibitor (IκB) was observed in both groups of old rats, but with a greater significance after CR treatment (24 mAL vs. 6 mAL, *p < 0.05; 24 mCR *vs*. 6 mAL, ^$$^*p* < 0.01; Figure 4A). Increment in IκB phosphorylation leads to its degradation and consequent activation of NFκB. Although the aging and CR treatment had no effect on NFκB protein level in the cytoplasm, its level was increased in nuclear fraction of old rats regardless of the diet regime (24 mAL vs. 6 mAL, *p < 0.05; 24 mCR vs. 6 mAL, ^$$$^*p* < 0.001), with a more severe increment of inflammatory response after CR compared with aged rats on AL diet (24 mCR vs. 24 mAL, ^#^*p* < 0.05; Figure 4B). The level of proinflammatory cytokine *Tnf*α was also significantly increased in aged rats on AL diet and after restricted diet regime (24 mAL vs. 6 mAL, *p < 0.05; 24 mCR vs. 24 mAL, ^#^*p* < 0.05; 24 mCR vs. 6 mAL, ^$^*p* < 0.05; Figure 4D). Interestingly, only the interaction between CR and aging led to increased *Tlr4* gene expression in the liver of old rats after late-onset CR (24 mCR vs. 24 mAL, ^##^*p* < 0.01; 24 mCR vs. 6 mAL, ^$$^*p* < 0.01; Figure 4C).

### Late-Onset Calorie Restriction Lowers the Level of Corticosterone and Its Nuclear Receptor in the Liver

**FIGURE 5:**
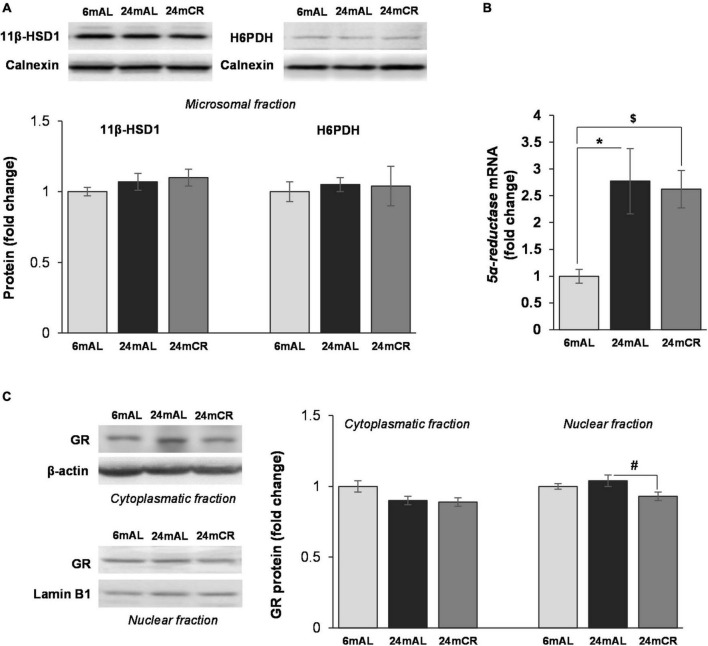
Glucocorticoid prereceptor metabolism and GR protein level in the liver. Representative Western blots and relative quantification of 11β-HSD1 and H6PDH protein levels in microsomal fraction **(A)** and GR protein levels in cytoplasmatic and nuclear fraction **(C)** of the liver of adults (6 mAL), aged rats on AL diet (24 mAL), and old rats on CR (24 mCR). Calnexin, β-actin and Lamin B1 were used as loading controls for microsomal, cytoplasmatic, and nuclear fraction, respectively. Quantification of *5*α*-reductase* mRNA **(B)** level in the liver was done relative to the amount of β-actin. All values are given as means ± SEM (*n* = 8). Asterisk indicates significant difference between aged rats on AL diet and adults (24 mAL vs. 6 mAL, *p < 0.05) and symbol $ indicates a significant difference between old rats on CR and adults fed AL (24 mCR *vs*. 6 mAL, ^$^*p* < 0.05).

Protein level of 11β-HSD1 and the level of enzyme that provides its cofactor, H6PDH, were unchanged in all the examined groups (Figure 5A). However, the level of corticosterone in the liver was increased in aged rats on AL diet as compared with adults, while CR treatment significantly lowered its level (24 mAL vs. 6 mAL, *p < 0.05; 24 mCR vs. 24 mAL, ^#^*p* < 0.05; Table 1). Aging process stimulated glucocorticoid clearance in the liver, as *5*α*-reductase* mRNA level was increased in both groups of old animals (24 mAL vs. 6 mAL, *p < 0.05; 24 mCR vs. 6mAL, ^$^*p* < 0.05; Figure 5B). As a result of *5*α*-reductase* overexpression and decreased corticosterone concentration, the protein level of GR in the nuclear fraction of the liver was decreased in aged rats on CR regime as compared to old animals on AL diet (24 mCR vs. 24 mAL, ^#^*p* < 0.05; Figure 5C).

## Discussion

On the one hand, the results from this study showed that late-onset CR has some positive effects on age-related homeostasis disturbances, as it leads to reduced body weight, VAT and liver mass, and serum triglyceride level. In addition, restrictive diet regime induces the activity of AMPK, suppresses *de novo* FA synthesis, stimulates β-oxidation, decreases lipotoxicity, and limits triglyceride synthesis and packaging in the liver. On the other hand, inflammatory response associated with aging becomes more severe after late-onset CR, which could be ascribed to inability of old rats to restore the potential for antioxidant protection in the setting of stimulated FA oxidation. Late-onset CR also reduced corticosterone concentration and increased *5*α*-reductase* expression in the livers of aged rats limiting the hormone’s ability to activate the GR. This probably results in diminution of anti-inflammatory properties of glucocorticoids after late-onset CR and additionally contributes to the deterioration of immune response in the liver of old rats.

One of the most evident age-induced alterations of standard-fed old animals from this study is weight gain. Increased body and adipose tissue mass could be referred to as an anorexigenic response caused by age-related leptin resistance and progressive imbalance between fat storage and lipolytic processes in adipocytes (22, 23). Age-induced adiposity increases the level of circulating FFAs and limits glucose utilization that burdens liver’s efforts to maintain glucose tolerance and insulin sensitivity (24, 25). This is in agreement with the increased hepatic *Gck* expression and decreased blood glucose level observed in old animals in our study. Long-term *Gck* overexpression and hepatic glucose excess also observed in older mice and humans are associated with an elevated rate of *de novo* lipogenesis, increased hepatic triglyceride content, and circulating lipid levels, which contribute to the ectopic lipid accumulation and hypertriglyceridemia (26, 27). In this study, age-related shift of fat metabolism toward lipogenesis in old rats was confirmed by several findings: (1) increased ACC protein content, which implies stimulated initial step of *de novo* FA synthesis, (2) *Mttp* overexpression, which indicates enhanced triglyceride packaging, and (3) decreased level of CPT1 protein, as a hallmark of lower rate of FA β-oxidation. Observed increased FA influx *via* the CD36 transporter and lowered *Scd1* expression in old rats have further worsened liver lipid status due to accumulation of saturated FA that promotes hepatocellular lipotoxicity, injury, and apoptosis (28).

Reduction of body fat in older individuals is one of the essential benefits of energy-depleting conditions caused by a restricted diet regime (29). Old rats from the present study had a preserved ability to adequately respond to reduced caloric intake, as stimulatory phosphorylation of AMPK, crucial energy sensor in the liver, was increased. In this line, stimulated AMPK activity raised the inhibitory phosphorylation of ACC, thus leading to its repression, which resulted in the inhibition of FA *de novo* synthesis and stimulation of energy-producing processes such as β-oxidation (30). Indeed, it has been previously shown that ACC knockout mice are characterized by intensified β-oxidation in the muscle, heart, and liver, reduced body weight, and decreased fat content, even after significant caloric overload (31). In our study, redirection of lipid metabolism toward FA oxidation in aged rats after CR was demonstrated by an increased level of transcriptional coactivator PGC1α followed by partial increment of CPT1. Although the CPT1 expression was increased after CR, it could not reach protein level observed in adult rats. This could be explained by reduced oxidative capacity, previously observed in older people (32). Utilization of FA in β-oxidation after CR decreased the abundance of substrate for triglyceride synthesis and packing. It has been previously shown that increased AMPK activity is accompanied with inhibition of glycerol-3-phosphate acyltransferase, the first enzyme in the formation of glycerolipids (33). After CR, triglyceride packaging and secretion in the form of VLDL were most probably diminished, as transcription of genes encoding *Mttp* and *Apo B-100* was decreased and restored to the level observed in adult animals. The beneficial effects of CR are additionally proven by the significantly higher expression of *Scd1*, even in regard to adults, leading to the removal of saturated FA and its transformation into neutral lipid droplets in the liver (34). Hence, late-onset CR not only inhibits lipogenesis and stimulates β-oxidation but also leads to the reduction of hepatic lipotoxity, implying that applied diet regime was able to restore age-related imbalance of fat metabolism.

Impairment of lipid metabolism during aging is associated with increased FFAs and accumulation of cytotoxic lipids, which could act as ligands and second messengers that activate several protein kinases and trigger downstream proinflammatory signaling cascade (35). Although the level of FFAs was increased in standard-fed aged rats from this study, the expression of *Tlr4* receptor was unchanged. It was previously shown in macrophages that the aging process could even reduce the level of most TLRs and hence weaken their function (36, 37). However, FFAs can stimulate proinflammatory transcriptional regulator NFκB through initiation of Bax protein translocation into lysosomes resulting in release of cysteine protease and cathepsin B, potent activators of IκB kinase (38). In this study, increased ratio between phosphorylated and total IκB, which implies IκB ubiquitination and its subsequent degradation, was observed in aged animals and was more prominent after CR treatment. The lower level of NFκB inhibitor leads to its activation and translocation to the nucleus, which is in agreement with a higher level of NFκB protein observed in nuclear fraction and enhanced transcription of *Tnf*α gene in older rats. Interestingly, the activation of NFκB proinflammatory signaling pathways in aged rats on standard diet was further enhanced in rats exposed to late-onset CR. Other authors reported that a different percentage of restricted calorie regime: mild (15%), moderate (40%), and even higher (50%), lowered cytokine expression and led to the alleviation of inflammatory status (39–41). However, these CR treatments started earlier and had different duration implying that aggravation of inflammatory response observed in our study could rather be a consequence of postponed CR start point. An important factor in NFκB activation during aging could also be oxidative stress caused by increased availability of ROS, potent activators of redox-sensitive transcription factors, cytokines, and inflammasomes (42). Decreased expression of hepatic antioxidant enzymes SOD and catalase in our study confirmed impaired ability of old rats to maintain redox balance. One of the most expected outcomes of restricted diet regime is oxidative stress reduction. However, many studies reported that, despite limited oxidative damage, such dietary approach had a poor effect on mitochondrial ROS production or antioxidant enzyme activity (12). Similarly, in our study, the level of antioxidant enzymes in aged rats after late-onset CR remained decreased, also implying that their expression was not affected by the change in dietary regime. The inability of aged rats to adapt the expression of antioxidant enzymes to increased mitochondrial β-oxidation caused by CR could lead to excess of ROS. This can be, at least partially, the reason for significantly deteriorated inflammatory reaction observed in old rats on restrictive diet regime. Furthermore, increased expression of *Tlr4* receptor after late-onset CR could provoke additional proinflammatory signaling pathway. It has been previously shown that limited food access upregulated TLR4 in colonic cells and macrophages, thus leading to elevated production of TNFα (43). However, aggravated inflammatory response after late-onset CR found herein is examined only in male rats and represents a limitation of the present study. Indeed, previous literature data points to important sex-related alterations in rodent response to CR (44). Namely, it has been shown that different types of CR initiate modified proinflammatory response in female rats (45), implying that additional studies on female rats should be performed in order to adjust dietary protocols and therapeutic strategies.

Stressful conditions induced by aging and reduced caloric intake are suitable for action of glucocorticoid hormones. As the level of proteins involved in tissue-specific hormone regeneration remains unchanged, increased corticosterone concentration in the liver of standard-fed old rats was probably caused by hormone overflow from circulation. Age-induced chronic cytokine production could trigger corticosterone secretion and maintain its elevated level in the circulation due to dysfunction of negative feedback of the hypothalamic–pituitary–adrenal (HPA) axis (46, 47). In spite of increased corticosterone, the level of cytoplasmic GR and its translocation to the nucleus were not changed by aging. The lack of GR activation should be attributed to the increased expression of *5*α*-reductase* with aging that inactivates corticosterone to its tetrahydro metabolites, which can bind to GR but cannot lead to its dimerization and nuclear translocation (48). Although many studies have demonstrated a stimulatory effect of energy restriction on HPA axis activity in obese humans and animals (49), in this study, we observed a decrease of liver corticosterone and its restoration to the control level. This is in accordance with studies that showed a decrease in circulating corticosterone concentrations following 6 weeks of food deprivation in obese rats or following a 4-week severely energy-restricted diet in obese male and female rats (50, 51). The lower corticosterone level in the liver and retention of increased expression of *5*α*-reductase* resultantly lead to decreased GR level in the nuclear fraction, which implies the reduced GR signaling in the liver of old animals after CR. This can lead to the attenuation of anti-inflammatory properties of glucocorticoids and further progression of inflammatory response induced by aging. This assumption is confirmed by profound activation of NFκB and significantly increased *Trl4* and *Tnf*α gene expression in the liver of old animals on a restrictive diet regime. The possible mechanism could lay in diminished interaction between GR and NFκB and reduced expression of glucocorticoid-induced leucine zipper (GILZ) that attenuates NFκB translocation to the nucleus (52).

## Conclusion

In conclusion, short duration and late-onset CR exerts beneficial effect on lipid content, but has a negative impact on hepatic inflammatory status. This implies that the type of the diet could contribute to age-related chronic diseases progression in older individuals and must be chosen carefully. Reduction of lipogenic nutrients and supplementation with antioxidants and vitamins and minerals that have anti-inflammatory properties are recommended. Consumption of foods that promote gut microbiome diversity and composition could be especially beneficial because it reduces the level of bacterial lipopolysaccharides that provoke inflammatory response *via* TLR4 receptor. Only targeted, balanced, and mild regimens of CR with an appropriate duration and start point could promote longevity and optimal conditions for healthy aging.

## Data Availability Statement

The raw data supporting the conclusions of this article will be made available by the authors, without undue reservation.

## Ethics Statement

The animal study was reviewed and approved by the Ethical Committee for the Use of Laboratory Animals of the Institute for Biological Research “Siniša Stanković” - The National Institute of Republic of Serbia, University of Belgrade (reference number 02-12/17).

## Author Contributions

AT, NV, and AD planned the experimental research. AM designed animal model. MV, AT, and MP carried out the experiments. AT, NV, and DVM analyzed the data. AT and MV wrote the manuscript with support from NV, AD, DVM, and AM. All authors reviewed and approved the final manuscript.

## Conflict of Interest

The authors declare that the research was conducted in the absence of any commercial or financial relationships that could be construed as a potential conflict of interest.

## Publisher’s Note

All claims expressed in this article are solely those of the authors and do not necessarily represent those of their affiliated organizations, or those of the publisher, the editors and the reviewers. Any product that may be evaluated in this article, or claim that may be made by its manufacturer, is not guaranteed or endorsed by the publisher.

## References

[1] Niccoli T Partridge L. Ageing as a risk factor for disease. *Curr Biol.* (2012) 22:R741–52. 10.1016/j.cub.2012.07.024 22975005

[2] Barzilai N Huffman DM Muzumdar RH Bartke A. The critical role of metabolic pathways in aging. *Diabetes.* (2012) 61:1315–22. 10.2337/db11-1300 22618766 PMC3357299

[3] Anderson RM Weindruch R. Metabolic reprogramming, caloric restriction and aging. *Trends Endocrinol Metab.* (2010) 21:134–41. 10.1016/j.tem.2009.11.005 20004110 PMC2831168

[4] Kim IH Kisseleva T Brenner DA. Aging and liver disease. *Curr Opin Gastroenterol.* (2015) 31:184–91. 10.1097/MOG.0000000000000176 25850346 PMC4736713

[5] Salminen A Kaarniranta K Kauppinen A. Age-related changes in AMPK activation: role for AMPK phosphatases and inhibitory phosphorylation by upstream signaling pathways. *Ageing Res Rev.* (2016) 28:15–26. 10.1016/j.arr.2016.04.003 27060201

[6] Boudaba N Marion A Huet C Pierre R Viollet B Foretz M. AMPK re-activation suppresses hepatic steatosis but its downregulation does not promote fatty liver development. *EBioMedicine.* (2018) 28:194–209. 10.1016/j.ebiom.2018.01.008 29343420 PMC5835560

[7] Ma L Wang R Wang H Zhang Y Zhao Z. Long-term caloric restriction activates the myocardial SIRT1/AMPK/PGC-1α pathway in C57BL/6J male mice. *Food Nutr Res.* (2020) 64:1–7. 10.29219/fnr.v64.3668 32082101 PMC7007760

[8] Jeon S-M. Regulation and function of AMPK in physiology and diseases. *Exp Mol Med.* (2016) 48:e245–245. 10.1038/emm.2016.81 27416781 PMC4973318

[9] Cantó C Auwerx J. PGC-1alpha, SIRT1 and AMPK, an energy sensing network that controls energy expenditure. *Curr Opin Lipidol.* (2009) 20:98–105. 10.1097/MOL.0b013e328328d0a4 19276888 PMC3627054

[10] Rogero MM Calder PC. Obesity, inflammation, toll-like receptor 4 and fatty acids. *Nutrients.* (2018) 10:432. 10.3390/nu10040432 29601492 PMC5946217

[11] Boden G. Fatty acid-induced inflammation and insulin resistance in skeletal muscle and liver. *Curr Diab Rep.* (2006) 6:177–81. 10.1007/s11892-006-0031-x 16898568

[12] Walsh ME Shi Y Van Remmen H. The effects of dietary restriction on oxidative stress in rodents. *Free Radic Biol Med.* (2014) 66:88–99. 10.1016/j.freeradbiomed.2013.05.037 23743291 PMC4017324

[13] Rao G Xia E Nadakavukaren MJ Richardson A. Effect of dietary restriction on the age-dependent changes in the expression of antioxidant enzymes in rat liver. *J Nutr.* (1990) 120:602–9. 10.1093/jn/120.6.602 2352034

[14] Kharwanlang B Sharma R. Glucocorticoid hormones in aging. In: Rattan S Sharma R editors. *Hormones in Ageing and Longevity. Healthy Ageing and Longevity.* (Vol. 6), Cham: Springer (2017). 10.1007/978-3-319-63001-4_3

[15] Dallman MF la Fleur SE Pecoraro NC Gomez F Houshyar H Akana SF. Minireview: glucocorticoids—food intake, abdominal obesity, and wealthy nations in 2004. *Endocrinology.* (2004) 145:2633–8. 10.1210/en.2004-0037 15044359

[16] White PC Rogoff D McMillan DR Lavery GG. Hexose 6-phosphate dehydrogenase (H6PD) and corticosteroid metabolism. *Mol Cell Endocrinol.* (2007) 265–266:89–92. 10.1016/j.mce.2006.12.022 17240046 PMC1852482

[17] Stewart MJ Parikh S Xiao G Tonge PJ Kisker C. Structural basis and mechanism of enoyl reductase inhibition by triclosan. *J Mol Biol.* (1999) 290:859–65. 10.1006/jmbi.1999.2907 10398587

[18] Todorovic ST Smiljanic KR Ruzdijic SD Djordjevic ANM Kanazir SD. Effects of different dietary protocols on general activity and frailty of male wistar rats during aging. *J Gerontol A Biol Sci Med Sci.* (2018) 73:1036–44. 10.1093/gerona/gly015 29415252 PMC6037071

[19] Prvulovic MR Milanovic DJ Vujovic PZ Jovic MS Kanazir SD Todorovic ST Late-onset calorie restriction worsens cognitive performances and increases frailty level in female wistar rats. *J Gerontol A Biol Sci Med Sci.* (2021) 77:glab353. 10.1093/gerona/glab353 34957511

[20] Teofiloviæ A Brkljaèiæ J Djordjevic A VojnoviæMilutinoviæ D Tappy L Matiæ G Impact of insulin and glucocorticoid signalling on hepatic glucose homeostasis in the rat exposed to high-fructose diet and chronic stress. *Int J Food Sci Nutr.* (2020) 71:815–25. 10.1080/09637486.2020.1728236 32070154

[21] Spector T. Refinement of the coomassie blue method of protein quantitation. A simple and linear spectrophotometric assay for less than or equal to 0.5 to 50 microgram of protein. *Anal Biochem.* (1978) 86:142–6. 10.1016/0003-2697(78)90327-5 655375

[22] Izquierdo AG Crujeiras AB Casanueva FF Carreira MC. Leptin, obesity, and leptin resistance: where are we 25 years later? *Nutrients.* (2019) 11:2704. 10.3390/nu11112704 31717265 PMC6893721

[23] Bonzón-Kulichenko E Moltó E Pintado C Fernández A Arribas C Schwudke D Changes in visceral adipose tissue plasma membrane lipid composition in old rats are associated with adipocyte hypertrophy with aging. *J Gerontol Ser A.* (2018) 73:1139–46. 10.1093/gerona/gly081 29668887

[24] Frohnert BI Jacobs DR Steinberger J Moran A Steffen LM Sinaiko AR. Relation between serum free fatty acids and adiposity, insulin resistance, and cardiovascular risk factors from adolescence to adulthood. *Diabetes.* (2013) 62:3163–9. 10.2337/db12-1122 23670973 PMC3749355

[25] Escrivá F Gavete ML Fermín Y Pérez C Gallardo N Alvarez C Effect of age and moderate food restriction on insulin sensitivity in Wistar rats: role of adiposity. *J Endocrinol.* (2007) 194:131–41. 10.1677/joe.1.07043 17592027

[26] Ferre T Riu E Franckhauser S Agudo J Bosch F. Long-term overexpression of glucokinase in the liver of transgenic mice leads to insulin resistance. *Diabetologia.* (2003) 46:1662–8. 10.1007/s00125-003-1244-z 14614559

[27] Flannery C Dufour S Rabøl R Shulman GI Petersen KF. Skeletal muscle insulin resistance promotes increased hepatic *de novo* lipogenesis, hyperlipidemia, and hepatic steatosis in the elderly. *Diabetes.* (2012) 61:2711–7. 10.2337/db12-0206 22829450 PMC3478531

[28] Alkhouri N Dixon LJ Feldstein AE. Lipotoxicity in nonalcoholic fatty liver disease: not all lipids are created equal. *Expert Rev Gastroenterol Hepatol.* (2009) 3:445–51. 10.1586/egh.09.32 19673631 PMC2775708

[29] Bales CW Porter Starr KN. Obesity interventions for older adults: diet as a determinant of physical function. *Adv Nutr.* (2018) 9:151–9. 10.1093/advances/nmx016 29659687 PMC5916429

[30] Hardie DG Pan DA. Regulation of fatty acid synthesis and oxidation by the AMP-activated protein kinase. *Biochem Soc Trans.* (2002) 30:1064–70. 10.1042/bst0301064 12440973

[31] Srivastava RAK Pinkosky SL Filippov S Hanselman JC Cramer CT Newton RS. AMP-activated protein kinase: an emerging drug target to regulate imbalances in lipid and carbohydrate metabolism to treat cardio-metabolic diseases. *J Lipid Res.* (2012) 53:2490–514. 10.1194/jlr.R025882 22798688 PMC3494254

[32] Layec G Haseler LJ Richardson RS. Reduced muscle oxidative capacity is independent of O2 availability in elderly people. *Age.* (2013) 35:1183–92. 10.1007/s11357-012-9442-6 22760857 PMC3705121

[33] Ruderman N Prentki M. AMP kinase and malonyl-CoA: targets for therapy of the metabolic syndrome. *Nat Rev Drug Discov.* (2004) 3:340–51. 10.1038/nrd1344 15060529

[34] Turyn J Stojek M Swierczynski J. Up-regulation of stearoyl-CoA desaturase 1 and elongase 6 genes expression in rat lipogenic tissues by chronic food restriction and chronic food restriction/refeeding. *Mol Cell Biochem.* (2010) 345:181–8. 10.1007/s11010-010-0571-x 20721682

[35] Geng Y Faber KN de Meijer VE Blokzijl H Moshage H. How does hepatic lipid accumulation lead to lipotoxicity in non-alcoholic fatty liver disease? *Hepatol Int.* (2021) 15:21–35. 10.1007/s12072-020-10121-2 33548031 PMC7886759

[36] Renshaw M Rockwell J Engleman C Gewirtz A Katz J Sambhara S. Cutting edge: impaired toll-like receptor expression and function in aging. *J Immunol.* (2002) 169:4697–701. 10.4049/jimmunol.169.9.4697 12391175

[37] Boehmer ED Goral J Faunce DE Kovacs EJ. Age-dependent decrease in Toll-like receptor 4-mediated proinflammatory cytokine production and mitogen-activated protein kinase expression. *J Leukoc Biol.* (2004) 75:342–9. 10.1189/jlb.0803389 14634059

[38] Feldstein AE Werneburg NW Canbay A Guicciardi ME Bronk SF Rydzewski R Free fatty acids promote hepatic lipotoxicity by stimulating TNF-alpha expression via a lysosomal pathway. *Hepatol Baltim Md.* (2004) 40:185–94. 10.1002/hep.20283 15239102

[39] Park CY Park S Kim MS Kim H-K Han SN. Effects of mild calorie restriction on lipid metabolism and inflammation in liver and adipose tissue. *Biochem Biophys Res Commun.* (2017) 490:636–42. 10.1016/j.bbrc.2017.06.090 28630003

[40] Allen BD Liao C-Y Shu J Muglia LJ Majzoub JA Diaz V Hyperadrenocorticism of calorie restriction contributes to its anti-inflammatory action in mice. *Aging Cell.* (2019) 18:e12944. 10.1111/acel.12944 30938024 PMC6516174

[41] MacDonald L Hazi A Paolini AG Kent S. Calorie restriction dose-dependently abates lipopolysaccharide-induced fever, sickness behavior, and circulating interleukin-6 while increasing corticosterone. *Brain Behav Immun.* (2014) 40:18–26. 10.1016/j.bbi.2014.01.005 24440143

[42] Chung HY Kim DH Lee EK Chung KW Chung S Lee B Redefining chronic inflammation in aging and age-related diseases: proposal of the senoinflammation concept. *Aging Dis.* (2019) 10:367–82. 10.14336/AD.2018.0324 31011483 PMC6457053

[43] Belmonte L Achamrah N Nobis S Guérin C Riou G Bôle-Feysot C A role for intestinal TLR4-driven inflammatory response during activity-based anorexia. *Sci Rep.* (2016) 6:35813. 10.1038/srep35813 27779218 PMC5078809

[44] Kane AE Sinclair DA Mitchell JR Mitchell SJ. Sex differences in the response to dietary restriction in rodents. *Curr Opin Physiol.* (2018) 6:28–34. 10.1016/j.cophys.2018.03.008 31231711 PMC6588196

[45] Dogan S Ray A Cleary MP. The influence of different calorie restriction protocols on serum pro-inflammatory cytokines, adipokines and IGF-I levels in female C57BL6 mice: short term and long term diet effects. *Meta Gene.* (2017) 12:22–32. 10.1016/j.mgene.2016.12.013 28373962 PMC5375115

[46] Herman JP McKlveen JM Ghosal S Kopp B Wulsin A Makinson R Regulation of the hypothalamic-pituitary-adrenocortical stress response. *Compr Physiol.* (2016) 6:603–21. 10.1002/cphy.c150015 27065163 PMC4867107

[47] Gupta D Morley JE. Hypothalamic-pituitary-adrenal (HPA) axis and aging. *Compr Physiol.* (2014) 4:1495–510. 10.1002/cphy.c130049 25428852

[48] McInnes KJ Kenyon CJ Chapman KE Livingstone DEW Macdonald LJ Walker BR 5alpha-reduced glucocorticoids, novel endogenous activators of the glucocorticoid receptor. *J Biol Chem.* (2004) 279:22908–12. 10.1074/jbc.M402822200 15044432

[49] Seimon RV Hostland N Silveira SL Gibson AA Sainsbury A. Effects of energy restriction on activity of the hypothalamo-pituitary-adrenal axis in obese humans and rodents: implications for diet-induced changes in body composition. *Horm Mol Biol Clin Investig.* (2013) 15:71–80. 10.1515/hmbci-2013-0038 25436734

[50] Dubuc PU Carlisle HJ. Food restriction normalizes somatic growth and diabetes in adrenalectomized ob/ob mice. *Am J Physiol.* (1988) 255:R787–93. 10.1152/ajpregu.1988.255.5.R787 3056042

[51] Hainer V Stich V Kunesová M Parízková J Zák A Wernischová V Effect of 4-wk treatment of obesity by very-low-calorie diet on anthropometric, metabolic, and hormonal indexes. *Am J Clin Nutr.* (1992) 56:281S–2S. 10.1093/ajcn/56.1.281S 1615903

[52] Ronchetti S Migliorati G Riccardi C. GILZ as a mediator of the anti-inflammatory effects of glucocorticoids. *Front Endocrinol.* (2015) 6:170. 10.3389/fendo.2015.00170 26617572 PMC4637413

